# NeuroTrace 500/525 identifies human induced pluripotent stem cell-derived brain pericyte-like cells

**DOI:** 10.1186/s13041-021-00893-5

**Published:** 2022-01-10

**Authors:** Seo Young Kim, Jihye Choi, Junhee Roh, Chul Hoon Kim

**Affiliations:** 1 Department of Pharmacology, BK21 PLUS Project for Medical Science, Brain Research Institute, Yonsei University College of Medicine, 03722 Seoul, Korea; 2 Kyung Hee University College of Medicine, 02447 Seoul, Korea

**Keywords:** Pericytes, hiPSCs, NeuroTrace 500/525

## Abstract

In the CNS, pericytes are important for maintaining the blood–brain barrier (BBB) and for controlling blood flow. Recently, several methods were suggested for the differentiation of human pluripotent stem cells (hPSCs) into brain mural cells, specifically pericytes or vascular smooth muscle cells (vSMCs). Unfortunately, identifying the pericytes from among such hPSC-derived mural cells has been challenging. This is due both to the lack of pericyte-specific markers and to the loss of defining anatomical information inherent to culture conditions. We therefore asked whether NeuroTrace 500/525, a newly developed dye that shows cell-specific uptake into pericytes in the mouse brain, can help identify human induced pluripotent stem cell (hiPSC)-derived brain pericyte-like cells. First, we found that NeuroTrace 500/525 specifically stains primary cultured human brain pericytes, confirming its specificity in vitro. Second, we found that NeuroTrace 500/525 specifically labels hiPSC-derived pericyte-like cells, but not endothelial cells or vSMCs derived from the same hiPSCs. Last, we found that neuroectoderm-derived vSMCs, which have pericyte-like features, also take up NeuroTrace 500/525. These data indicate NeuroTrace 500/525 is useful for identifying pericyte-like cells among hiPSC-derived brain mural cells.

**Supplementary Information:**

The online version contains supplementary material available at 10.1186/s13041-021-00893-5.

Pericytes are vascular mural cells (VMCs) that surround endothelial cells. They support vessel stability, blood flow regulation, angiogenesis, and inflammation [1]. The blood vessel coverage by pericytes is highest in the CNS [2]. Brain pericytes ensheathe the capillary endothelium and contribute to BBB integrity by working with specialized endothelial cells, astrocytic end-feet, and neurons in structures called neurovascular units [1]. It has been difficult to study pericytes because of a lack of pericyte-specific marker molecules. Neural/glial antigen 2 (NG2) and platelet-derived growth factor receptor β (PDGFRβ) are often used as pericyte markers, but both are also expressed in vSMCs, another VMC subtype [3]. Recently, NeuroTrace 500/525 was reported as an exclusive marker for pericytes in the mouse brain [4]. It was originally used to identify neurons in fixed brain tissues, but when administered to the brains of *Pdgfrβ-Cre*; *tdTomato* transgenic mice, the VMCs of which express tdTomato, NeuroTrace 500/525 labeled only tdTomato-positive cells ensheathing capillaries (pericytes). It did not label the ring-like cells that line pre-capillaries or arterioles (vSMCs) [4]. This pericyte-specific uptake of NeuroTrace 500/525 suggests the presence of molecular transport mechanisms exclusive to pericytes. Although these results are promising, the utility of NeuroTrace 500/525 has not yet been tested in human cells, which may have different transport mechanisms, or in in vitro culture systems that lack many of the non-autologous effects of in vivo systems.

There are various protocols for differentiating hiPSCs into cell types of interest. Recently, differentiation protocols to produce human VMCs were developed, making it possible to produce human pericyte-like cells or vSMCs in culture [5–8]. Making human pericytes in vitro will surely improve the relevance of disease modeling and drug screening work related to pericytes. The problem is that both hiPSC-derived pericyte-like and vSMC cells express NG2 and PDGFRβ without any anatomical information that would otherwise, in live mice, help discriminate these two mural cell types. Thus, we asked whether NeuroTrace 500/525 could help us discern pericyte-like cells differentiated from hiPSCs.

To determine whether NeuroTrace 500/525 specifically labels human pericytes in vitro, we added the dye to various types of primary cultured cells. We found green fluorescence only in human primary cultured brain pericytes, indicating that NeuroTrace 500/525 exclusively labels human pericytes in culture conditions (Fig. 1A). Next, we co-differentiated the same hiPSC line into endothelial cells and pericyte-like cells via neural crest stem cells (NCSCs), which are the embryonic precursor to forebrain mural cells [9] (Fig. 1B, D; see Additional file 1 for the detailed methods). We observed that as the NCSCs progressed to pericyte-like cells, their expression of the NCSC marker p75^NTR^ fell, while their expression of NG2 and PDGFRβ increased (Fig. 1B, Additional file 2: Fig. S1). Among DAPI-positive cells, 95.9 ± 1.2% were NG2-positive and 97.1 ± 0.7% were PDGFRβ–positive. We also found pericyte-like cells can self-assemble with endothelial cells in an endothelial cord forming assay in vitro and promote the formation of longer vascular cords (Fig. 1C). These data suggest hiPSC-derived pericyte-like cells possess the key attributes of brain pericytes. After adding NeuroTrace 500/525 to hiPSC-derived NCSCs, endothelial and pericyte-like cells, we found that the dye specifically stains pericyte-like cells (Fig. 1D and 97.2 ± 1.2% of DAPI-positive cells were NeuroTrace-positive).

Next, we differentiated the same hiPSC line into pericyte-like cells and vSMCs to determine whether NeuroTrace 500/525 can discriminate these two VMC subtypes. We noted that both cell types express NG2 and PDGFRβ, ruling out these markers for the discrimination of pericytes from vSMCs (Fig. 1E; Additional file 2: Fig. S2). Interestingly, we found only vSMCs express αSMA, suggesting that the pericyte-like cells we produced may recapitulate capillary pericytes [10] (Fig. 1E). When we added NeuroTrace 500/525, we found that these capillary pericyte-like cells exhibit clear and selective uptake (97.7 ± 2.1% of DAPI-positive cells were NeuroTrace-positive), whereas vSMCs did not exhibit any dye uptake (Fig. 1E).

Next, we focused on two protocols designed to differentiate hiPSCs into vSMCs. The main difference between the two approaches is that the first one induces vSMCs directly from neuroectodermal cells (vSMCs-NE) [7], while the second one induces vSMCs via NCSCs (vSMCs-NCSC) [8]. vSMCs-NE reportedly have enough pericyte-like properties to support capillary structures [11]. Furthermore, the molecular signature of vSMCs-NCSC is more like that of human brain vSMCs [8]. We therefore wondered whether NeuroTrace 500/525 can discriminate vSMCs-NCSC and vSMCs-NE. We split and differentiated the same hiPSC line into vSMCs-NCSC and vSMCs-NE [7, 8] (Fig. 1F). Immunostaining revealed that vSMCs-NE express NG2, PDGFRβ and αSMA (Fig. 1F). We were surprised to find that NeuroTrace 500/525 also labels vSMCs-NE (Fig. 1G and 96.4 ± 0.5% of DAPI-positive cells were NeuroTrace-positive). To determine whether vSMCs-NE have pericyte-like attributes, we performed a Matrigel-based endothelial cord forming assay by co-culturing HUVECs with vSMCs-NCSC or vSMCs-NE. We found vSMCs-NE co-cultured with HUVECs contribute to forming longer capillary cords; the vSMCs-NCSC seem to pull the growing cords apart due to their contractile properties (Fig. 1H, I). Because vSMCs-NE are not as potent as NCSC-derived pericyte-like cells in supporting endothelial cord formation, they may represent a transition state between vSMCs and mature pericytes. We also confirmed the specificity of NeuroTrace 500/525 in cells derived from a different hiPSC line [12] (Additional file 2: Fig. S3).

**Fig. 1:**
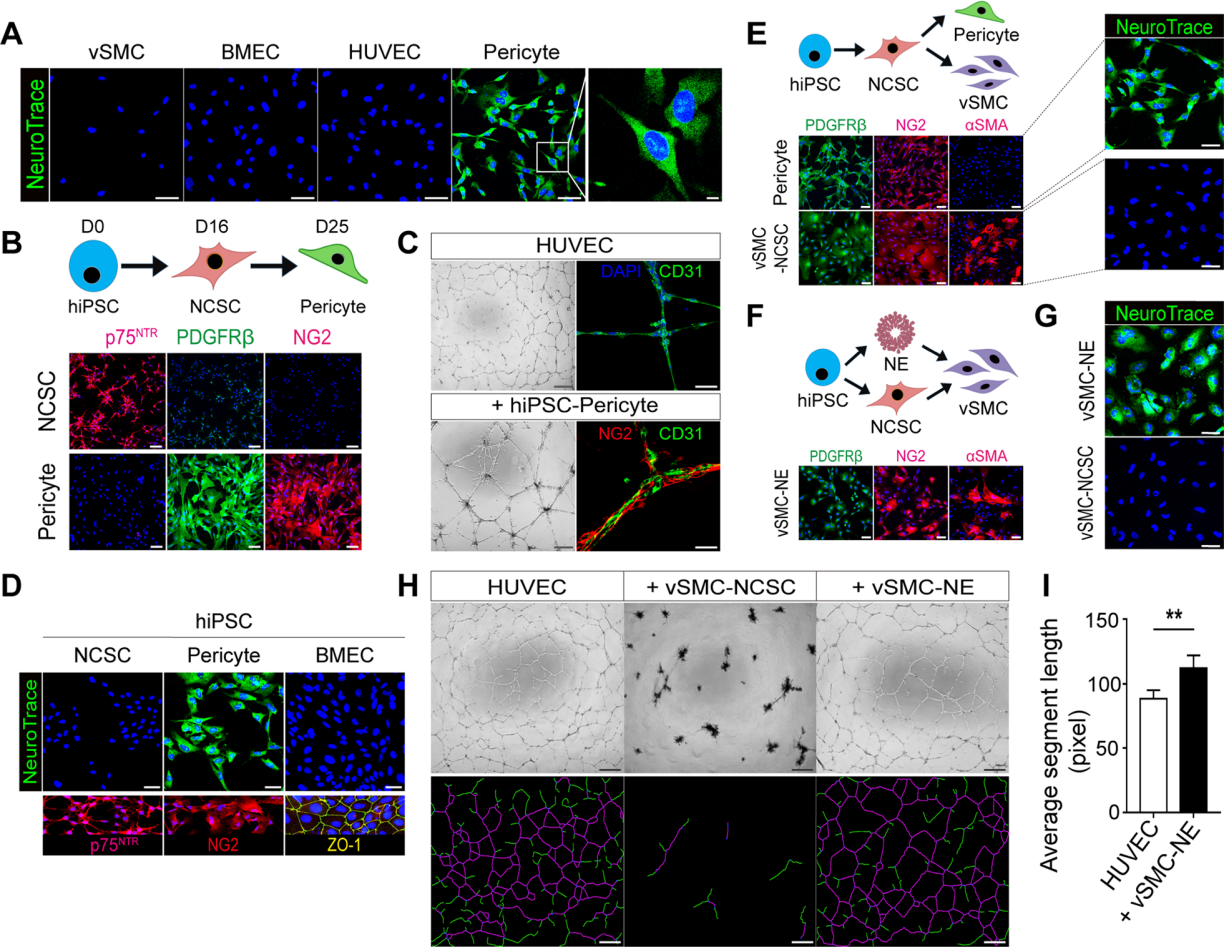
NeuroTrace 500/525 selectively labels human brain pericyte-like cells in vitro. **A** NeuroTrace 500/525 staining was performed on human umbilical vein endothelial cells (HUVECs), human primary cultured brain vSMCs, brain microvascular endothelial cells (BMECs), and brain pericytes. Cellular uptake of NeuroTrace 500/525 appears as green fluorescence. Scale bars, 50 μm (5 μm in magnified area). **B** Schematic representation of pericyte differentiation. Brain pericyte-like cells were differentiated from hiPSCs (ASE9209) through a neural crest intermediate. Immunostaining images reveal changes in the expression of the NCSC marker p75^NTR^ and the pericyte markers PDGFRβ and NG2. Scale bars, 100 μm. **C** In vitro endothelial cord formation assay using HUVECs and hiPSC-derived brain pericyte-like cells. Representative bright field images of HUVECs alone or HUVECs co-cultured with hiPSC-derived pericyte-like cells. Scale bars, 500 μm. Immunostaining images of HUVECs (stained with CD31 antibodies, green) alone or HUVECs co-cultured with hiPSC-derived pericyte-like cells (stained with NG2 antibodies, red). Scale bars, 50 μm. **D** NeuroTrace 500/525 staining of hiPSC-derived NCSCs, pericyte-like cells, and BMECs. hiPSC-derived NCSCs, pericyte-like cells, and BMECs were incubated with NeuroTrace 500/525 (upper panels) for 20 min. hiPSC-derived NCSCs, pericyte-like cells, and BMECs were stained with antibodies against cell type-specific markers such as p75^NTR^ (magenta), NG2 (red), and ZO-1 (green), respectively (lower panels). Scale bars, 50 μm. **E** Schematic representation of pericyte-like cells and vSMCs differentiation from the same hiPSCs (ASE9209) via a common NCSC intermediate. Immunostaining of mural cell markers (i.e., PDGFRβ, NG2, and αSMA). Scale bars, 100 μm. NeuroTrace 500/525 staining of NCSC-derived pericyte-like cells and vSMCs (vSMCs-NCSC). Scale bars, 50 μm. **F** Schematic representation of neuroectoderm-derived vSMCs (vSMCs-NE) and NCSC-derived vSMCs (vSMC-NCSC) differentiations from the same hiPSCs (ASE9209). Immunostaining analysis of mural cell markers (i.e., PDGFRβ, NG2 and αSMA) in both cell types. Scale bars, 100 μm. **G** NeuroTrace 500/525 staining of vSMCs-NE and vSMCs-NCSC. Scale bars, 50 μm. **H** In vitro endothelial cord formation assay using HUVECs, vSMCs-NE, and vSMCs-NCSC. Representative bright field images (upper panels) and maps of cord networks analyzed by Angiogenesis Analyzer (lower panels) showing segments (magenta) and branches (green). Scale bars, 500 μm. **I** Quantification of average segment length from bright field images of vascular cord networks formed by HUVECs alone or HUVECs co-cultured with vSMCs-NE. *p*-values were calculated using an unpaired Student’s *t* test. Results are presented as means ± SEM. ** *p* < 0.01 (*n* = 5 for each group). All microscopy images are representative of at least three independent experiments

These data suggest NeuroTrace 500/525 is a useful marker for the discrimination of hiPSC-derived pericyte-like cells. To our knowledge, NeuroTrace 500/525 is the only way to selectively label pericytes among vascular cells in vitro and in vivo in both human and rodents, suggesting a universal mechanism across species. NeuroTrace 500/525 enters neither neurons nor glia in mouse brains [4]. Considering this selectivity, it is unlikely that NeuroTrace 500/525 stains neural cells of human origin. Hence, it is plausible that the transport system for NeuroTrace 500/525 comprises molecules expressed in pericytes of both humans and rodents. Thus, although NeuroTrace 500/525 has proven to be a useful marker of pericytes, its utility will likely be extended when the molecules that transport it into pericytes are identified. Their discovery will pave the way to the development of pericyte-specific antibodies and even a Cre recombinase line that will greatly expedite pericyte research.

## Supplementary Information

**Additional file 1.** Materials and methods.

**Additional file 2.** Figure S1–S3.

## Data Availability

All data and materials are available upon request.

## Abbreviations

hiPSCs: Human induced pluripotent stem cells
vSMCs: Vascular smooth muscle cells
NCSCs: Neural crest stem cells
NE: Neuroectoderm
