# Supplementary material for: NeuroTrace 500/525 identifies human induced pluripotent stem cell-derived brain pericyte-like cells
Source: Mol Brain. 2022 Jan 10;15:11. doi: 10.1186/s13041-021-00893-5 (PMC8751259; doi:10.1186/s13041-021-00893-5)
Supplement: Supplementary file 1 — Additional file 1. Materials and methods. [file 13041_2021_893_MOESM1_ESM.docx]

**Materials and Methods**

**Primary cell culture**

Human brain vascular pericytes and human brain vascular smooth muscle cells were purchased from ScienCell Research Laboratories (Carlsbad, CA, USA). They were plated onto poly-L-lysine-coated plates and maintained in pericyte medium containing 2% FBS and pericyte growth supplement or smooth muscle cell medium containing 2% FBS and smooth muscle cell growth supplement (ScienCell), respectively. This study used only pericytes and smooth muscle cells between passages 3 and 6. Human brain microvascular endothelial cells (BMECs) were purchased from Cell Systems (Kirkland, WA, USA), plated onto fibronectin-coated plates, and maintained in complete classic medium containing serum and CultureBoost^TM^ (Cell Systems) between passages 3 and 5. Human umbilical vein endothelial cells (HUVECs) were purchased from Lonza (Basel, Switzerland) and cultured in EGM^TM^-2 endothelial cell growth media.

**Differentiation of pericyte-like cells from hiPSCs**

Our pericyte differentiation protocol was adapted from a recently published protocol [1]. Pericytes were differentiated from hiPSCs through a neural crest intermediate. For NCSC differentiation, ASE9209 (Applied StemCell, Milpitas, CA, USA) or IMR90-4 (WiCell Research Institute, Madison, WI, USA) cells were singularized using Accutase (STEMCELL Technologies, Vancouver, Canada) and seeded at 8.75 × 10^5^ cells/well in Matrigel-coated 6-well plates with mTeSR1 (STEMCELL Technologies) and 10 μM Y27632 (Tocris, Minneapolis, MN, USA). After a 24-hr incubation, the medium was switched to E6 (Gibco, Grand Island, NY, USA) supplemented with 22.5 μg/ml heparin sodium salt (Sigma-Aldrich, St Louis, MO), 1 μM CHIR99021 (Tocris), 10 μM SB431542 (Tocris), 10 ng/ml bFGF (R&D Systems, Minneapolis, MN, USA), and 1 μM dorsomorphin (Sigma-Aldrich). The cells were then differentiated for 15 days by changing the medium (E6-CSFD) daily. During NCSC differentiation, when the cells reached 100% confluence, the differentiating neural crest cells were passaged at a 1:6 ratio. Closing in on day 15, the cells were passaged at a 1:2 or 1:3 ratio. On day 15, the cells (10^7^ cells) were resuspended in 60 μl of buffer containing 0.5% bovine serum albumin (BSA) and 2 mM EDTA in DPBS (without Ca^2+/^Mg^2+^). Then, 20 μl of NCSC magnetic microbeads (conjugated to p75^NTR^ antibodies) and 20 μl of FcR blocking reagent (Miltenyi Biotec, Bergisch Gladbach, Germany) were added. After a 15-min incubation at 4°C, cells labeled with anti-p75 ^NTR^ NCSC microbeads were sorted through MS columns using a MiniMACS magnetic separator (Miltenyi Biotec) according to the manufacturer’s instructions. After MACS, the sorted p75 ^NTR^+ NCSCs were replated onto 6-well plates at 2 × 10^6^ cells/well in E6-CSFD with 10 μM Y27632. After a 24-hr incubation, the medium was switched to E6 medium supplemented with 10% FBS. The cells were further differentiated into pericytes for 9 days with daily changes of the E6-FBS medium.

**Differentiation of brain microvascular endothelial cells (BMECs) from hiPSCs**

Our BMEC differentiation protocol was adapted from a previously published protocol [2]. First, hiPSCs were seeded at 2 × 10^5^ cells/well onto Matrigel-coated 6-well plates in mTeSR1 with 10 μM Y27632. The cells were maintained in mTeSR1 for 3 days. When the cells reached a density of 3 × 10^5^ ~ 5 × 10^5^ cells/well, the medium was switched to unconditioned medium (UM) containing DMEM/Ham's F12 (Gibco) with 20% Knockout Serum Replacement (Gibco), 1× MEM nonessential amino acids (Gibco), 1 mM L-glutamine (Gibco), and 0.1 mM β-mercaptoethanol (Sigma-Aldrich) for 6 days. On day 6, the medium was switched from UM to endothelial cell (EC) medium supplemented with 10 μM retinoic acid (Sigma-Aldrich). EC medium is composed of human endothelial serum-free medium (hESFM, Gibco), 20 ng/ml bFGF, and 1% platelet-poor plasma-derived human serum (Sigma-Aldrich). On day 8, the cells were dissociated with Accutase and seeded onto collagen/fibronectin-coated plates. The plates were coated with a 4:1:5 ratio of collagen, fibronectin, and water and incubated for at least 1 hr at 37°C. The cells were then cultured in EC medium for 3 days.

**NCSC-derived vSMC (vSMC-NCSC) differentiation**

Our vSMC-NCSC differentiation protocol was adapted from a previously published protocol [3]. First, hiPSCs were seeded at 8.75 × 10^5^ cells/well onto Matrigel-coated 6-well plates with mTeSR1 and 10 μM Y27632. After a 24-hr incubation, the medium was switched to E6-CSFD. The hiPSCs were differentiated toward NCSCs for 14 days by changing the E6-CSFD medium daily. On day 16, the NCSCs were sorted via MACS, and the resulting p75^+^ NCSCs were replated onto 6-well plates at 2 × 10^5^ cells/well in E6-CSFD with 10 μM Y27632. After another 24-hr incubation, the medium was switched to E6-PT, E6 medium supplemented with 10 ng/ml PDGF-BB (Peprotech, Rocky Hill, New Jersey, USA) and 2 ng/ml TGF-β1 (Peprotech). The cells were differentiated into vSMCs-NCSC for 12 days with daily changes of the E6-PT medium.

**Neuroectoderm-derived vSMC (vSMC-NE) differentiation**

Our vSMC-NE differentiation protocol was adapted from a previously published protocol [4]. First, hiPSCs were passaged onto Matrigel-coated 6-well plates at 10–20 clusters/well in mTeSR1. After a 24-hr incubation, the hiPSCs were cultured in E6 medium supplemented with 10 μM SB431542 and 12 ng/ml bFGF for 5 days. Then, the cells were subcultured onto new Matrigel-coated plates at a density of 2 × 10^5^ ~ 3 × 10^5^ cells/well. On day 6, the medium was switched to E6-PT, and the cells were further differentiated into vSMCs-NE in E6-PT for 12 days.

**Immunocytochemistry**

Cells were fixed for 15 min at room temperature with 4% paraformaldehyde (PFA) and then washed three times with PBS. Then, the cells were blocked for 1 hr in blocking buffer at room temperature and incubated overnight at 4°C with primary antibodies. Antibodies and staining conditions are listed in Table 1. The next day, the cells were washed three times with PBS and incubated with secondary antibodies (1:1,000 dilution). After a 1-hr incubation at room temperature on a rocking platform, the cells were incubated for 10 min with DAPI (Thermo Fisher Scientific, Rutherford, NJ, USA) diluted 1:1,000 in PBS. The cells were washed three times with PBS and mounted in a mounting solution. Images were taken on an Olympus fluorescence microscope or a Zeiss confocal microscope.

**Table 1. List of primary antibodies and staining conditions**

| **Antibodies** | **Source** | **Identifier** | **Staining solution** | **Dilution** |
| --- | --- | --- | --- | --- |
| p75^NTR^ | Advanced Targeting System | AB-N07 | 1% BSA | 1:500 |
| NG2 | Millipore | MAB2029 | 5% goat serum + 0.4% TX-100 | 1:100 |
| PDGFRβ | CST | 3169 | 5% goat serum + 0.4% TX-100 | 1:100 |
| αSMA | Abcam | ab7817 | 5% non-fat dry milk + 0.4% TX-100 | 1:200 |
| ZO-1 | Invitrogen | 40-2200 | 5% goat serum + 0.4% TX-100 | 1:100 |
| CD31 | Abcam | ab32457 | 5% goat serum + 0.4% TX-100 | 1:200 |

**RNA extraction and quantitative real time PCR (RT-PCR)**

Cells were collected in TRIzol Reagent (Thermo Fischer Scientific). Then, cDNAs were synthesized from RNA samples using the PrimeScript™ 1st strand cDNA Synthesis Kit (TAKARA, Shiga, Japan) according to the manufacturer’s instructions. Gene expression levels of pericytes and smooth muscle cells were tested by quantitative real-time PCR (RT-PCR) using SYBR Green Master Mix (Applied Biosystems, Waltham, MA, USA) on a StepOne Real-Time PCR system (Applied Biosystems). The primers used in this study were as follows: *NGFR* F’-GTGGGACAGAGTCTGGGTGT; *NGFR* R’- AAGGAGGGGAGGTGATAGGA; *PDGFRβ* F’- GCTCACCATCATCTCCCTTATC; *PDGFRβ* R’-CTCACAGACTCAATCACCTTCC; *ACTA2* F’-TGTTCCAGCCATCCTTCATC; and *ACTA2* R’- GCAATGCCAGGGTACATAGT. *CSPG4* is a predesigned probe (Hs. PT. 58.39417158) from Integrated DNA Technologies (IDT, Newark, NJ, USA).

**NeuroTrace 500/525 staining**

Cells were incubated with NeuroTrace 500/525 (Invitrogen) diluted 1:2,000 in E6 medium for 20 min at 37°C. Then, the cells were fixed with 4% PFA for 15 min, washed with PBS, and incubated for 10 min with DAPI diluted 1:1,000 in PBS at room temperature. Finally, the cells were mounted in a mounting solution (Invitrogen), and images were taken on an Olympus fluorescence microscope or a Zeiss confocal microscope.

***In vitro* endothelial cord formation assays and their quantification**

Eight-well glass chamber slides were coated with 200 μl of Matrigel/well and incubated for 1 hr at 37°C to solidify the Matrigel. HUVECs were plated at 4.4×10^4^ cells/well in 500 μl of EGM2 medium alone or with 2.2×10^4^ hiPSC-derived pericyte-like cells, vSMCs-NCSC, or vSMCs-NE. The cells were incubated for 24 hrs at 37°C, and bright-field images were taken before fixation. Then, the cords were fixed for 15 min at room temperature with 4% PFA and stained according to the immunocytochemistry methods described above. The cords were mounted onto glass slips and imaged using a Zeiss confocal microscope. In a defined area of interest, Angiogenesis Analyzer (ImageJ software) represents vascular cord structures as vectorial objects and provides a quantitative evaluation of their junctions, branches, segments. Angiogenesis Analyzer calculates average segment length as the sum of the lengths of all segments (binary line linked with two junctions) divided by the total number of the segments.

**Statistical analysis**

All data analysis was conducted with GraphPad Prism version 7 (GraphPad Software, Inc., La Jolla, CA, USA). *P*-values were calculated via an unpaired Student’s *t* test or one-way analysis of variance (ANOVA) followed by multiple comparisons with Bonferroni correction. All data are presented as means ± standard error of the mean (SEM). The significance level was set at *p* < 0.05.

**References**

1. Stebbins MJ, Gastfriend BD, Canfield SG, Lee MS, Richards D, Faubion MG, et al. Human pluripotent stem cell-derived brain pericyte-like cells induce blood-brain barrier properties. Sci Adv. 2019;5(3):eaau7375.

2. Lippmann ES, Azarin SM, Kay JE, Nessler RA, Wilson HK, Al-Ahmad A, et al. Derivation of blood-brain barrier endothelial cells from human pluripotent stem cells. Nat Biotechnol. 2012;30(8):783-91.

3. Cheung C, Goh YT, Zhang J, Wu C, Guccione E. Modeling cerebrovascular pathophysiology in amyloid-beta metabolism using neural-crest-derived smooth muscle cells. Cell Rep. 2014;9(1):391-401.

4. Cheung C, Bernardo AS, Trotter MW, Pedersen RA, Sinha S. Generation of human vascular smooth muscle subtypes provides insight into embryological origin-dependent disease susceptibility. Nat Biotechnol. 2012;30(2):165-73.
