# Supplementary material for: NeuroTrace 500/525 identifies human induced pluripotent stem cell-derived brain pericyte-like cells
Source: Mol Brain. 2022 Jan 10;15:11. doi: 10.1186/s13041-021-00893-5 (PMC8751259; doi:10.1186/s13041-021-00893-5)
Supplement: Supplementary file 2 — Additional file 2. Figure S1–S3. [file 13041_2021_893_MOESM2_ESM.docx]

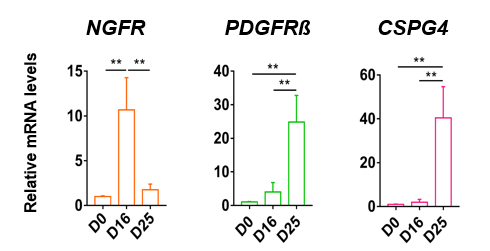


**Figure S1**. Changes in the mRNA levels of molecular markers for NCSCs and pericytes during pericyte-like cell differentiation via NCSCs from hiPSCs. A quantitative real-time PCR revealed the mRNA expression of the neural crest marker gene *NGFR* (encoding p75^NTR^) and the mural cell marker genes *PDGFRβ* (encoding PDGFRβ) and *CSPG4* (encoding NG2) at day 0 (D0) (hiPSC stage), D16 (NCSC stage), and D25 (pericyte-like cell stage). The mRNA expression level of each gene was normalized to the reference gene *GAPDH*. *P*-values were calculated using the one-way ANOVA followed by Bonferroni *post hoc* tests for multiple comparisons. *NGFR:* F (2,6) = 19.36, *p* =0.0024, *PDGFRβ*: F (2,6) = 21.10, *p* =0.0019, *CSPG4*: F (2,6) = 22.20, *p* =0.0017. Results are presented as means ± SEM of three independent experiments. ** *p* < 0.01.


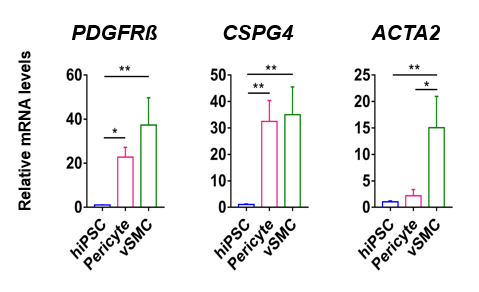


**Figure S2**. The mRNA levels of mural cell markers in hiPSC-derived pericyte-like and vSMC cells. A quantitative RT-PCR analysis shows the mRNA expression levels of the mural cell marker genes *PDGFRβ, CSPG4,* and *ACTA2*. The mRNA expression level of each gene was normalized to the reference gene *GAPDH*. *P*-values were calculated using the one-way ANOVA followed by Bonferroni *post hoc* tests for multiple comparisons. *PDGFRβ*: F (2,6) = 17.30, *p* =0.0032, *CSPG4*: F (2,6) = 18.69, *p* =0.0026, *ACTA2*: F (2,6) = 14.89, *p* =0.0047. Results are presented as means ± SEM of three independent experiments. * *p* < 0.05, ** *p* < 0.01.


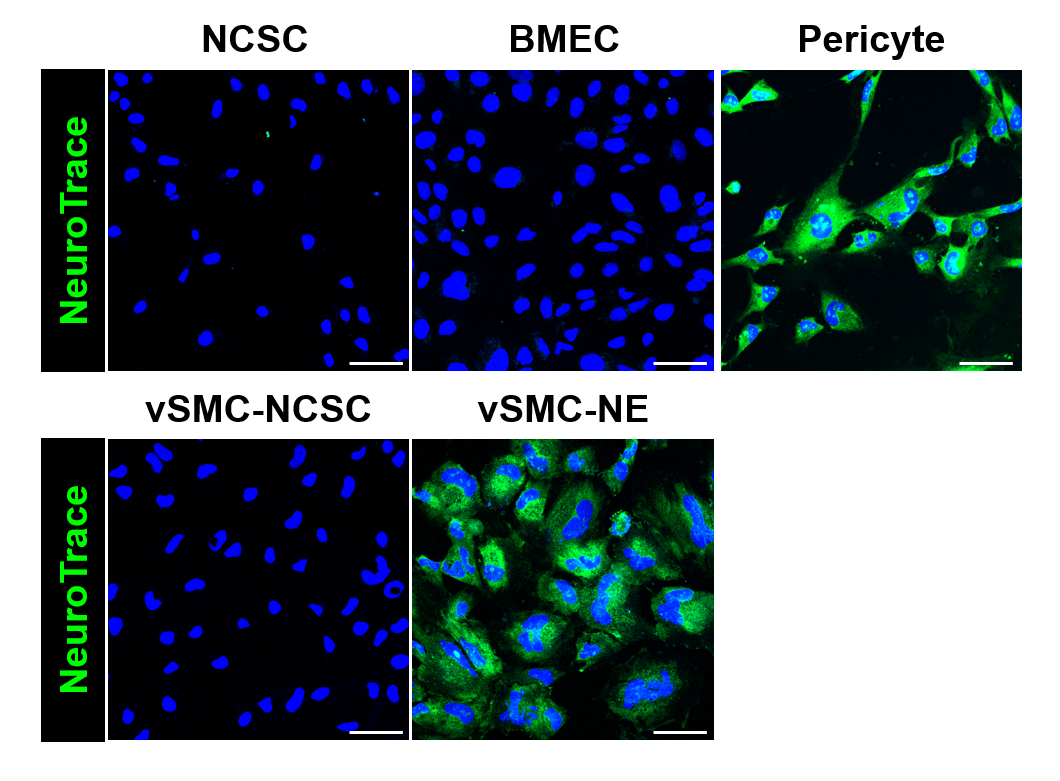


**Figure S3.** The selectivity of NeuroTrace 500/525 is exhibited also in cells derived from a different hiPSC-line (IMR90-4). We differentiated IMR90-4 cells into NCSCs, BMECs, pericyte-like cells, vSMCs-NCSC, and vSMCs-NE. NeuroTrace 500/525 labeled IMR90-4-derived pericyte-like cells and vSMCs-NE. All images are representatives of three independent experiments. Scale bars, 50 μm.
